# Organisational culture and trust as influences over the implementation of equity-oriented policy in two South African case study hospitals

**DOI:** 10.1186/s12939-017-0659-y

**Published:** 2017-09-15

**Authors:** Ermin Erasmus, Lucy Gilson, Veloshnee Govender, Moremi Nkosi

**Affiliations:** 1 Health Policy and Systems Division, School of Public Health and Family Medicine University of Cape Town, Rondebosch, South Africa; 2 Health Policy and Systems Division, School of Public Health and Family Medicine, University of Cape Town, Rondebosch, South Africa; 3 Department of Global Health and Development, Faculty of Public Health and Policy London School of Hygiene and Tropical Medicine, London, UK; 4 Alliance for Health Policy and Systems Research, World Health Organization, Geneva, Switzerland; 5 Medscheme, Cape Town, South Africa

**Keywords:** Organisational culture, Organisational trust, Policy implementation, Equity, South Africa, Hardware, Software, People-centeredness, Patients’ rights charter, Uniform patient fee schedule

## Abstract

**Background:**

This paper uses the concepts of organisational culture and organisational trust to explore the implementation of equity-oriented policies – the Uniform Patient Fee Schedule (UPFS) and Patients’ Rights Charter (PRC) - in two South African district hospitals. It contributes to the small literatures on organisational culture and trust in low- and middle-income country health systems, and broader work on health systems’ people-centeredness and “software”.

**Methods:**

The research entailed semi-structured interviews (Hospital A *n* = 115, Hospital B *n* = 80) with provincial, regional, district and hospital managers, as well as clinical and non-clinical hospital staff, hospital board members, and patients; observations of policy implementation, organisational functioning, staff interactions and patient-provider interactions; and structured surveys operationalising the Competing Values Framework for measuring organisational culture (Hospital A *n* = 155, Hospital B *n* = 77) and Organisational Trust Inventory (Hospital A *n* = 185, Hospital B *n* = 92) for assessing staff-manager trust.

**Results:**

Regarding the UPFS, the hospitals’ implementation approaches were similar in that both primarily understood it to be about revenue generation, granting fee exemptions was not a major focus, and considerable activity, facility management support, and provincial support was mobilised behind the UPFS.

The hospitals’ PRC paths diverged quite significantly, as Hospital A was more explicit in communicating and implementing the PRC, while the policy also enjoyed stronger managerial support in Hospital A than Hospital B.

Beneath these experiences lie differences in how people’s values, decisions and relationships influence health system functioning and in how the nature of policies, culture, trust and power dynamics can combine to create enabling or disabling micro-level implementation environments.

**Conclusions:**

Achieving equity in practice requires managers to take account of “unseen” but important factors such as organisational culture and trust, which are key aspects of the organisational context that can profoundly influence policies. In addition to implementation “hardware” such as putting in place necessary staff and resources, it emphasises “software” implementation tasks such as relationship management and the negotiation of values, where equity-oriented policies might be interpreted as challenging health workers’ status and values, and paying careful attention to how policies are practically framed and translated into practice, to ensure key equity aspects are not neglected.

## Background

People-centered health systems (PCHS), a concept that has gained currency in global health policy and systems scholarship recently [1–3], has two faces. The first is *normative* and promotes values associated with health system equity goals, such as participatory governance, and the equal treatment of people. The second is *descriptive* and recognises that people’s decisions are central to health systems, health systems can operate only through relationships between varied actors, and values are key to health system actors’ decisions and actions. In this descriptive sense, people’s values, decisions and actions are key to health system functioning, even if they do not live up to the normative sense of the concept. Both faces of PCHS emphasise, therefore, the "software" dimensions of health systems, i.e. the human, social and political factors [4–6], that are critical in organisational capacity (to make decisions, undertake tasks, do things differently [7]), and that influence the achievement of social change and equity goals [8].

Against the background of these current global debates, this paper addresses a common question asked by health policymakers, managers and researchers: why are policies often implemented in ways that diverge from policy objectives and intended changes? [4, 5, 9, 10, 11]. The study it reports examined the implementation of the Uniform Patient Fee Schedule (UPFS) and Patients’ Rights Charter (PRC) in South Africa, in 2006–7.

The contemporary relevance of this study for South Africa lies partly in the specific policies considered. Like the contemporary, proposed South African National Health Insurance (NHI) reforms, that seek to achieve and deepen universal health coverage (UHC), the UPFS and PRC required changes in how hospitals and clinics function in order to promote equity [12]. Past South Arican experience has also well demonstrated that implementing equity-oriented policies often generates unexpected and sometimes unwanted health policy implementation outcomes, such as creating rather than removing access barriers, experiencing resistance to equity-promoting actions, and undermining rather than strengthening the motivation of health workers [13-16].

A recent systematic review has noted that the body of empirical research about policy implementation in low- and middle-income countries (LMICs) is still limited [10]. Although it includes some focus on street-level bureaucrats [17], only limited attention has been paid to features of the organisational settings that theory suggests influence their behaviour – such as organisational culture and organsisational trust [9, 18, 19]. Indeed, to the extent that current LMIC literature addresses either organisational culture or trust, it only maps organisational cultures, relates them to concerns such as job satisfaction and quality improvement initiatives [20] or considers how trust in the provider-patient relationship influences interactions, service quality and responsiveness [2, 21].

The study, therefore, set out to investigate the experience of policy implementation and, more specifically, the influence of street-level bureaucrats, organisational culture and organisational trust over the implementation of the South African UPFS and PRC policies. Street-level bureaucrats (SLBs) are frontline policy implementers who have regular and direct interaction with the recipients of government services and the power to exercise some discretion over the services, benefits and sanctions recipients receive [9]. Organisational culture was understood as artefacts, values and assumptions that are to some extent shared by members of an organisation and that influence organisational functioning [18]; and organisational trust refers to trust between different people and parts of an organisation, in this case staff and managers [22]. Both organisational dimensions shape SLB behaviour [9, 23].

## Methods

### Study design features

This research adopted a nested case study design, for two reasons. First, a case study is a way of inquiring empirically about a phenomenon in its “real-life” context when the context is expected to have a major impact on the phenomenon [24]. This was relevant as we sought to investigate the implementation of the UPFS and PRC as it unfolded in the “real worlds” of two hospitals, with the assumption that contextual software such as organisational culture and trust would have a major impact on the implementation process. Second, case studies are particularly suited to answering “how” and “why” questions [24], like ours: how were the UPFS and PRC policies implemented in practice in the case study hospitals, and why did implementation processes play out in these particular ways?

The primary case study unit was "the experience of implementing an equity-oriented health policy" and this was nested within the context of a case study hospital. Table 1 provides an overview of the two policies of focus. They were selected because they addressed different equity concerns and were quite different in nature. Our assumption was that these differences would illuminate the challenges faced in implementing equity-oriented policies, which seek to challenge the status quo, and would, more specifically, help to uncover values and exercises of power in implementation.

**Table 1:** Overview of the policies of focus

Uniform Patient Fee Schedule
After national-level approval, the UPFS was implemented across provinces in 2001/2. It sought to ensure the uniform billing of public hospital patients and stipulated that certain services, for example primary care and services to pregnant women and children younger than 6 years, were free of charge to all or almost all (members of health insurance schemes were, for example, excluded from certain free services). Other services were charged according to the patient’s income sources and levels and, where applicable, other factors such as health insurance membership and non-South African citizenship. Patients were classified as H0 (fully subsidised), H1 (highly subsidised), H2 (moderately subsidised), and H3 (full public sector rate) [46]. For exemptions and patient classification, H1 was the default category for a patient without sufficient proof of income. Providing documents from other government agencies, for example a card to prove receipt of a social grant or income from the Unemployment Insurance Fund (formal unemployment), would result in a move to H0. For other patients, especially the self-employed and those not regarded as formally unemployed, classification or exemption could involve making sworn statements at police stations to “prove” their unemployment (although this was not accepted by all facilities), completing an income and expenditure form, and providing proof of bills such as utility accounts that might shed light on their financial position.
Patients Rights Charter
The PRC, launched in the late 1990s, outlined to patients and health workers the common standards of service and behaviour expected. It was partly intended to rebalance the patient-health system relationship and to bring healthcare provision in line with South Africa’s new constitution, given that during the apartheid era “the vast majority of the South African population has experienced either a denial or violation of fundamental human rights, including rights to health care services” [47]. The PRC contained rights such as refusal of treatment, confidentiality and privacy, and a healthy and safe environment, which were balanced by responsibilities such as complying with prescribed treatment, taking care of health records, and respecting the rights of other patients and health workers.

The UPFS’ graduated fee levels and exemptions spoke to financial equity and access, while the PRC strove for acceptable care, equity through patient empowerment and the dignified treatment of all patients. The UPFS quite clearly delineated its key features and implementation requirements, but the PRC was much more open to interpretation in how it would be implemented. How can the right to confidentiality and privacy be protected when the layout of consulting rooms is not ideal? Given long travelling distances and health worker shortages, how can the right to a second opinion be effected? How can apparently competing issues, such as the right to refuse treatment and the responsibility to comply with prescribed treatment, be balanced? In practice, the PRC was commonly implemented through activities such as the distribution of pamphlets and posters, staff training, patient suggestion boxes, and providing staff with nametags. However, because rights and responsibilities must commonly be given practical effect in the patient-provider interaction, implementing the the PRC was always reliant on frontline workers’ discretion.

The two case study hospitals were, finally, selected to be relatively well-functioning as judged by local area managers, on the assumption that this approach would be conducive to drawing out positive implementation lessons. To limit the range of variation that might explain any differences in implementation experience, they were also similar in other dimensions - both were district hospitals, located in largely rural areas serving populations of relatively low socio- economic status. However, they were located in different provinces as dictated by the research institutes’ locations (full hospital details provided in the results section).

### Data collection: Approach and tools

Data collection was conducted in two phases each of around 2 months at the end of 2006 and then again the beginning of 2007; with a short break in-between for initial data analysis, debriefing, reflection, and further planning. The duration and intensity of the data collection, as well as the different tools used enabled rich insight into the research settings, as is needed in case study research. The short break between research phases also sought to reduce the burden on study participants.

As is common in case study work, mixed methods were used to examine UPFS and PRC implementation and assess organisational culture and organisational trust within the case study hospitals.

#### Qualitative data

Phase 1 included initial narrative interviews with a range of managers inside and outside the hospitals, as well as hospital staff, to understand key role-players and their descriptions of policy implementation processes; relationship mapping interviews within the hospitals to identify policy implementation networks and explore relationships across levels of the implementation chain; and observations of policy implementation, organisational functioning, staff interactions and patient-provider interactions within hospitals (Table 2). The observations continued in phase 2 and were supplemented by interviews with hospital board members and patients to gather insights into the experiences of additional stakeholders, as well as follow-up interviews with health workers and hospital managers to explore their perspectives on provider-patient relations and pick up on issues from the initial narrative interviews (Table 2).

**Table 2:** Qualitative interviews and respondents

Method	Respondents	Number of respondents	
		Hospital A	Hospital B
Initial narrative interviews	Provincial and regional managers, clinical and non-clinical hospital staff, hospital managers	47	27
Relationship mapping interviews	Hospital managers, clinical and non-clinical hospital staff	13	7
Further in-depth interviews (1)	Hospital board members, patients, district and provincial managers	25	28
Further in-depth interviews (2)	Hospital managers, clinical and non-clinical hospital staff	30	18

The key informants were purposively sampled, driven by the positions they occupied and the functions they fulfilled (giving different perspectives on the processes and questions the research sought to explore). One of the authors (MN) was primarily responsible for the interviews in Hospital A, while another (VG) was responsible for those in Hospital B, and both worked with a fieldworker. Most of the interviews, which generally lasted about an hour, were conducted in English, although the fieldworkers were able to conduct interviews in other languages, such as Afrikaans, Setswana and isiXhosa, if needed. The interviews were as far as possible recorded and transcribed (and translated into English if necessary). Where respondents did not want to be recorded, detailed interview notes were taken.

#### Quantitative data

Two structured self-assessment questionnaire surveys were also conducted, once we had built trust with hospital staff, to examine organisational culture and organisational trust, specifically (see Table 3). The questionnaires were widely distributed across the hospitals, participation was voluntary and anonymous and the intention was to get responses from as many staff members from different staff cadres as possible. We judged that, as a self-assessment questionnaire survey, the response rates were sufficient to provide a fair picture of the key trust and culture dynamics in the hospitals, especially in combination with the qualitative data. However, a higher response rate would have increased confidence in the quantitative results.

**Table 3:** Structured surveys and respondents

Method	Respondents	Number of respondents (response rate)	
		Hospital A: 481 staff	Hospital B: 193 staff
Organisational trust survey	Sample of all hospital staff	185 (38%)	92 (48%)
Organisational culture survey	Sample of all hospital staff	155 (32%)	77 (40%)

Based on the competing values framework (CVF) [25], the first survey examined the hospitals’ organisational culture. The CVF was initially derived from review of the organisational effectiveness literature [26], has been used in healthcare settings [27–29] and some work has been done to validate the related research instruments [30]. Considering these factors, our judgement was that it described general and recognisable organisational approaches and dilemmas that would be relevant in helping us understand the working of the South African case study hospitals.

**Fig. 1:**
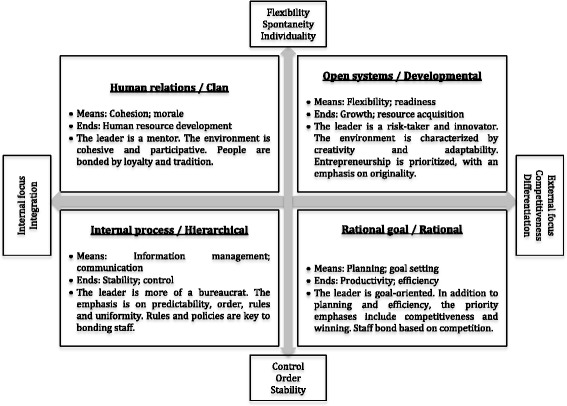
Organisational culture typology

The CVF proposes three value dimensions that combine into four organisational models, originally described as the human relations, open system, internal process and rational goal models [25], and subsequently labelled as clan, developmental, hierarchical and rational models [31-33], as illustrated in Fig. 1. The horizontal axis ranges from an internal focus that emphasises integration and the well-being of organisational members, to an external focus, more concerned with competitiveness, the well-being of the organisation itself and differentiation from other organisations; whilst the vertical axis ranges from organisational valuing of spontaneity, flexibility and individuality, to valuing order, control and stability. Third, organisations emphasise different means and ends.

The second survey explored organisational trust through the short form of the organisational trust inventory (OTI) [22]. This tool stems from work that explored the impact of trust on organisational functioning [19], was systematically developed and validated, and is intended to measure the trust between people within an organisation or between organisations. It consists of three sets of questions aimed at eliciting respondent views on managerial behaviours in terms of keeping commitments, not taking excessive advantage, and negotiating honestly. We used this instrument to help us explore trust between managers and others within the hospitals.

Finally, we used *both* the CVF and OTI to deepen our understanding of these key dimensions of the case study settings and complement our qualitative data. Whilst we recognised that organisational culture and trust might themselves be related (e.g. an organisational culture might incorporate certain values about who can be trusted or might lead to certain trust dynamics), the competing values framework did not clearly incorporate organisational trust and we felt it important to examine it separately.

### Data analysis

Following case study design principles [24], we initially prepared two separate case study reports that, drawing on all available data, presented holistic and detailed narratives of implementation experience around both policies in each hospital. We then compared and contrasted the two hospital reports to identify critical patterns of implementation experience. This paper, then, presents a synthesis of the experiences - rich descriptions structured to highlight critical issues.

Our data analysis approaches were the same for each case study. With respect to the qualitative material, we used the framework analysis approach common to policy studies, which combines deductive and inductive coding [34]. We generated initial codes from the research objectives and relevant concepts (such as organisational culture, organisational trust and discretionary power), but also added codes after an initial careful reading of the data. To establish consistency, the researchers first cross-coded some transcripts but each researcher was subsequently responsible for coding a selection of transcripts. Data were then extracted from transcripts into tables using the codes, and subsequently were grouped into broader themes. This thematic analysis then supported the development of the case study narrative report for each site. Early stages of the qualitative data analysis process also involved team debriefing meetings with the researchers who collected data; for example, in-between the two phases of data collection, as well as meetings to reflect collectively on the data and initial interpretrations.

The organisational culture survey required respondents to allocate weights to the various workplace descriptions contained in each question, with each description corresponding to one of the cultural types. In analysing these data, we summed and averaged these weights across survey questions to develop a representation of the distribution of clan, developmental, hierarchical and rational cultures in each hospital. Analysis of the organisational trust survey, meanwhile, involved calculating the percentage agreement/disagreement with each of the survey questions. Here we present the results organised by negative and positive management behaviours as this succinctly represents our overall judgement of the main trust dynamics in the case study hospitals.

The researchers also reflected together on the qualitative and quantitative data, to explore both how they complemented or contradicted each other and how to synthesise the information into narratives of the policy implementation experiences. Interpretive judgments – for example, concerning the influence of organisational culture and trust on policy implementation - were inevitable given the phenomena of focus in this research, but also presented the possibility of inappropriate judgements. As recommended for health policy and systems research [35, 36], collective reflection, therefore, sought to ensure the quality of the analysis by encouraging researchers to consider the assumptions they brought to the analysis and the different angles from which the data could be viewed. In addition, triangulation across data sources, research methods, and researchers [37], as well as cross-case analysis, underpinned the credibility of our account of the policy implementation experiences within the case study hospitals.

Finally, the use of theory in analysis, helping to guide the researchers in making sense of complex experiences and supporting the attempt to explicitly explain the patterns and processes of policy implementation, was a key strength of the analytic process [35].

The research received ethics clearance from the universities of Cape Town and the Witwatersrand, and was also approved by the relevant provincial health departments and hospital authorities prior to the study’s commencement.

## Results

### Understanding hospital settings and policy implementation experiences

#### The study settings

Hospital A was a Roman Catholic mission hospital before it was taken over by an apartheid “homeland” government in the 1970s. Under the racial segregationist system of the time, the South African government created “independent” territories or “homelands” for black ethnic groups. After the democratic transition of 1994, the hospital became the responsibility of a provincial government. At the time of the research, the hospital had 8 wards, 290 active beds, 481 staff members (561 approved posts) and an annual budget of approximately R58 million (2006: US$ ±8,12 million). It provided wide-ranging services (including surgical, paediatric, maternity, psychiatry, outpatient dental, physiotherapy and anti-retroviral therapy) to about 190,000 people scattered across almost 100 villages and residential areas. This hospital was located in the third most deprived local government area in the province. The overall level of unemployment was high. Subsistence and commercial farming were important economic activities, with many of the employed working as seasonal labourers and some being employed by government departments.

Hospital B had general, surgical, medical, paediatrics and maternity wards, as well as an outpatient department and casualty section. The hospital had 85 active beds, 193 staff members (222 approved posts) and an annual budget of R28 million (US$ ±3,92 million). The target population included the inhabitants of the local towns, which were up to 40 km away. As with Hospital A, this hospital was located in a comparatively deprived area where unemployment was high, approximately 8% of households were dependent upon social grants from government and where agriculture, and its associated seasonal employment, was the major economic activity.

#### Organisational culture

**Fig. 2:**
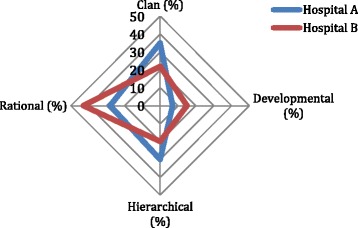
Organisational culture results: Hospitals A and B

Cohesion, participation and staff morale were important in Hospital A and being supportive of others was valued (Clan, 35%: Fig. 2). Qualitatively, this was reflected in respondents commonly referring to the hospital as a “family” or “home” and noting the close relationships among hospital staff, perhaps partially because of the many years that some staff members (particulalrly nurses) had worked at the hospital. This closeness is evidenced by managers’ accessibility, their comfortable interactions with other staff groups, and the participative style with which the core senior management group role-modelled approachability and inclusivity by drawing a broader group of unit managers from across the hospital into the facility’s day-to-day management. Co-existing with these values were significant orientations towards order, acting within rules and policies, and respecting reporting relationships (Hierarchical, 30%), as well as a strong competitive streak (Rational, 28%), a cultural element that is about performing well and achieving objectives. This showed, for example, in how the management team emphasised the awards the hospital had won for service delivery to rally support for revenue collection under the UPFS policy, reflecting a concern with the hospital’s good reputation and a desire to maintain it (interview data).

Compared to Hospital A, Hospital B was noticeable for its significant rational (43%), competitive and performance orientation. The hospital had, for example, won an award for its neatness and cleanliness. This standout cultural dimension was also observed qualitatively, for example in how staff valued the hospital’s public image and public recognition received from patients (observations and interview data), as was suggested by the display on a notice board of a patient letter praising the cleanliness of the facility and the good care received from staff, as well as staff comments about the regular positive reports about the hospital in the local newspaper and the satisfaction derived from this. With this important rational cultural feature came less of a clan (22%) and hierarchical (20%) orientation.

#### Organisational trust

**Fig. 3:**
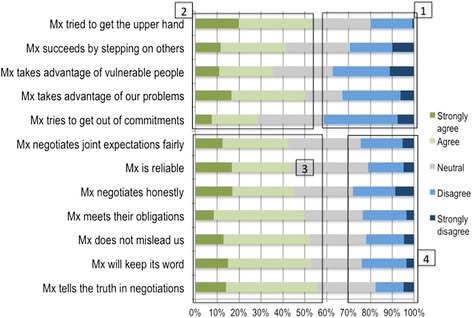
Trust in management in Hospital A

**Fig. 4:**
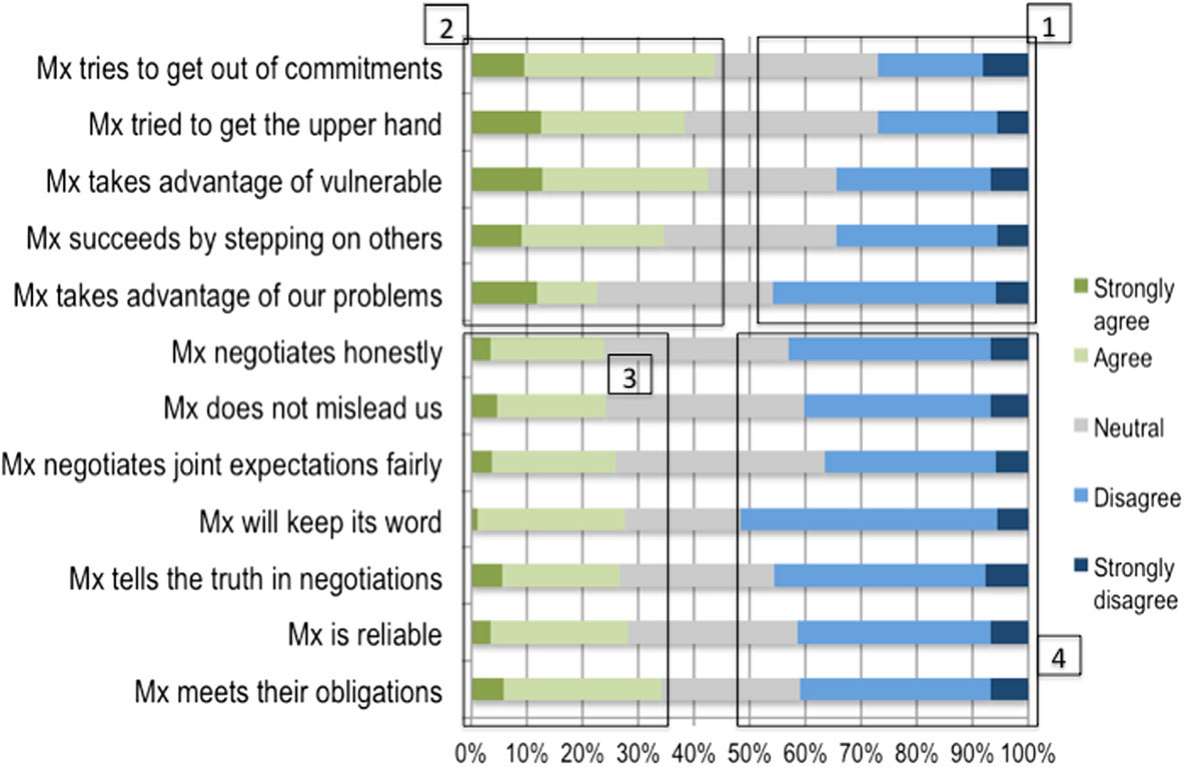
Trust in management in Hospital B

Perhaps reflecting organisational culture differences, and the particular importance of a clan culture, the staff in Hospital A appeared to have higher trust in their hospital management than in Hospital B (Figs. 3 and 4).

This is reflected in the differences between hospitals in survey responses on the positive management behaviours of negotiating joint expectations fairly, being reliable, negotiating honestly, meeting obligations, not misleading others, keeping promises and telling the truth in negotiations. There were much higher levels of agreement with these statements in Hospital A (indicating trust) than Hospital B (block 3), and much higher levels of disagreement in Hospital B (block 4).

Nonetheless, in both hospitals around a third of respondents disagreed with negative statements (indicating trust) about management behaviour regarding trying to get the upper hand, succeeding by stepping on others, taking advantage of vulnerable people, taking advantage of staff problems, and trying to get out of commitments (block 1). They also showed similar levels of agreement with these negative statements (indicating distrust) (block 2). Hospital A’s high levels of agreement with statements about trying to get the upper hand and taking advantage of staff problems were likely related to staff performance appraisals on-going at the time of the survey.

### Cross-hospital implementation experience: UPFS

The policies’ implementation approaches and experiences were similar across hospitals in some key respects, as revealed through observations and interviews.

#### Implementation practice

In both hospitals the UPFS was primarily understood by staff to entail revenue generation, with implementation success measured against a provincially determined annual target. Hospital A’s R800 000 target (US$ ±112,000) was perceived as somewhat arbitrary because it was set without consulting hospital managers or considering community unemployment. The target caused mixed feelings – managers expected staff to reach it, staff dreaded being seen as underperforming (less than half was actually collected), and also feared reaching it because it might then be increased further – and as a result, staff attempted to ensure payment by all patients able to pay. As it collected slightly more than the planned R1 641,000 (US$ ±229,831), the revenue target perhaps caused less anxiety in Hospital B, although managers clearly took steps to ensure patients did not slip through the payment net. For example, a case manager was appointed to recover fees by tracking the admission of full-paying patients, ensuring the documentation of procedures and medicines, checking health insurance authorisations for admissions, and ensuring correct accounts.

In contrast to revenue generation, granting fee exemptions was not a major focus of staff concern in either hospital, although patients who could not pay were not turned away. Most patients were allocated to a fee-paying category even if they did not have the necessary documents to secure an exemption, as they needed a classification to proceed to access care (Table 1); and many essentially became debtors, with few efforts to collect money from even those with long-standing debt, and much of the debt eventually written off. Observations and interviews suggested that the limited focus on exemptions was linked to:

- *Potentially complicated procedures for determining eligibility*, with patients required to present supporting documentation (Table 1);
- *The behaviour of clerks*, who rarely informed patients of the possibility of exemption; and
- *The knowledge of patients* who generally knew little about how fees practically applied to them, the possibility of exemption, who could get it and how.

#### Managerial support for the policy

In both hospitals considerable organisational activity, facility management support, and provincial support was mobilised behind the revenue target and UPFS. Provincial departments of health and finance supported the policy through, for example, training hospital staff and buying computers. In Hospital A, the organisational activity and managerial support included senior managers reminding staff to pay their own outstanding accounts, regular meetings between senior managers and administrators on UPFS implementation and revenue generation, a senior manager being continuously logged in to the billing system to monitor implementation, and hospital managers trying to have the revenue target reduced because of their failure to achieve it. In Hospital B, senior managers also supported the UPFS, an income and expenditure form was introduced, ostensibly to help indebted patients structure affordable repayments, and a case manager was appointed to ensure the recovery of fees.

#### Street-level bureaucrat behaviour

However, in one example of resistance against the case manager’s perceived encroachment on ward affairs and an increased administrative load, nurses reported, and were observed, not consistently completing the forms the case manager needed to ensure full billing, citing as reasons staff shortages, forgetting about the forms, the case manager not being sensitive enough to their time constraints, and that their primary responsibility was patient care, not administration. The strategy observed to counteract this resistance included the hospital chief executive accompanying the case manager on rounds. Table 4 then highlights other examples of street-level bureaucrat behaviour that influenced UPFS implementation across hospitals. It also shows that the researchers interpreted concerns such as not delaying the flow of patients, applying some “common sense” to their work, high workloads and frustration, and acting in support of the policy as explaining these behaviours.

**Table 4:** Street-level bureaucrat influences over UPFS implementation

	Behaviour	Rationale underpinning behaviour
Hospital A	Clerks rarely informed patients about the possibility of exemptions	Do not delay patient processing by activating difficult exemption processes
	Clerks occasionally broke the rules to exempt patients without proof of unemployment	Charging obviously unemployed patients from whom you will not recover money artificially inflates the outstanding amount shown in the financial system
	Clerks were sometimes rude to patients (as described by patients)	Long queues and frustration at patients not bringing the correct information that would make clerks’ job easier
	Medical staff turned back patients who sought care without first reporting to the clerks	Supporting policy implementation
Hospital B	Clerks sometimes used their discretion to classify patients without supporting documents, e.g. exempting patients clearly old enough to be pensioners or classifying patients familiar to the clerks	Applying some common knowledge and sense to the process
	Clerks sometimes classified patients declaring an income into a higher category than warranted by the declaration	Encouraging patients to bring supporting documents and ensuring they don’t cheat the system

Source: observations and in-depth interviews in each hospital; researcher judgements based on experience in each hospital

### Cross-hospital implementation experience: PRC

The hospitals’ PRC implementation paths diverged in important respects.

#### Implementation practice

Despite some patient complaints about the preferential treatment of certain patients, more caring staff attitudes in certain hospital sections, poor quality of care and disrespectful provider-patient interactions, the researchers’ observations in both hospitals were that staff broadly and overwhelmingly acted in line with the intention of the PRC in demonstrating respect and care for patients. However, beyond adherence in this general sense, a clear difference was that Hospital A to some extent explicitly implemented the PRC, but Hospital B did not.

The range of implementation activities identified through observation and interviews in Hospital A included PRC training in the early years of implementation, inclusion in a staff orientation programme (which had become less intense over time), the creation of an information desk and the use of staff in certain busy units as queue managers to facilitate service access, installing suggestion boxes, buying name tags for all staff to meet the requirement of being identified and named providers, and the widespread display of PRC posters. Interviews in Hospital B revealed the existence of quality assurance committees, the creation of a complaints mechanism and client surveys – all of which were primarily framed as general quality assurance, rather than being linked to the PRC. Also, most of the staff members in Hospital B were observed not to wear nametags and at the start of the research the PRC was not displayed, although a copy in English, one of three official languages, was later posted in a waiting room.

Staff in Hospital A also mentioned various ways in which the hospital had sought to communicate the PRC to the community, including the chief executive and senior staff appearing on a community radio station, speaking at church gatherings, attending tribal meetings and engaging in public meetings alongside traditional leaders and local government officials. Hospital B’s staff were silent on such initiatives. In both hospitals, although unexpectedly in Hospital A, patient awareness of the PRC ranged from poor to non-existent (patient interviews).

#### Managerial support for the policy

This difference in the explicit nature of PRC implementation between hospitals appeared to be underpinned by clearly differing management support for the PRC. The overall lack of support for the policy in Hospital B, and the underlying concerns, are clearly illustrated in this quote:

*“Nice in theory, but doesn’t work in practice. You will never see the PRC up in my hospital...there is an over-exposure to information...it is a difficult one, it is far too comprehensive, and even if we stick it on the wall people won’t read the first two sentences and they won’t know what it is about because it is complicated and convoluted and it is not something that we can adhere to. In 2003 we considered putting up the PRC and we didn’t have a Xhosa version and after that I felt let’s stick to Batho Pele. And also, casualty is so full of forms…there is an overload of information and people, even my staff, don’t read the notice boards. We should display a few core messages like where to get the contraceptive pill, which should be in bold language as simple as possible.”* (Hospital B, senior manager).

Yet both hospitals did implement the PRC, to some extent, indirectly. Hospital A was involved in an accreditation process implemented by an independent quality improvement and accreditation body, with some similar requirements to those of the PRC. In the case of Hospital B, the PRC was, at provincial, regional and facility levels, understood as just one aspect of quality of care and quality assurance. Indirect implementation occurred through managerial preference for Batho Pele (People First), a government-wide and non-health-specific quality improvement initiative that included principles that overlapped with the PRC, such as access, courtesy and citizens receiving full information about public services.

Across hospitals there was, however, less involvement and support for the PRC from higher authorities, compared to the UPFS. In Hospital A, respondents mostly felt the provincial and district offices played a small part in PRC implementation, not even clearly considering its implementation when visiting the hospital. Hospital A received a small number of PRC posters from the provincial office but then had to mobilise its own funds to print more and translate them from English into the local language. In Hospital B, meanwhile, no action was taken in response to the lack of PRC posters by the regional office, despite this being part of a checklist that it used for quarterly quality monitoring and evaluation.

#### Street-level bureaucrat behaviour

Nonetheless, across hospitals some staff were only grudging in their acceptance of the PRC - as demonstrated in common discourses about its risks for providers and in terms of patient behaviours (Table 5).

**Table 5:** Examples of grudging acceptance of the PRC

Discourse theme: The PRC does not adequately take account of health workers’ rights
“…it (PRC) gives the patients the right, you know, but on the other hand forgetting about the health providers…and at the end of the day we are the ones who are suffering…and at the end of the day we end up being rude to the patients, you know.” (Hospital A, nursing assistant)
Discourse theme: Patients know their rights, but not their responsibilities
“The challenge, I see the challenge mainly from the patients…they only look at their rights, but they forget that these rights, they go hand-in-hand with the responsibilities. The biggest challenge that we have is to maybe link the responsibilities to the rights because now everybody knows his right” (Hospital A, nurse) “They (patients) sometimes feel that they have the right to abuse us, but sometimes we feel neglected. If people are empowered, it comes with responsibility and I don’t think that people always realise it” (Hospital B, nurse)
Discourse theme: The PRC leaves providers open to abuse, with no recourse
“Patients can abuse the staff, but the staff can’t do anything. How much abuse can nurses take?” (Hospital B, nurse) “…because patients can walk in sometimes and really abuse and walk out of here with you not being able to say anything – which is what I think is not right” (Hospital A, doctor).

Source: interview data

On balance, and reflecting on the data and experiences holistically, our judgement was that this grudging acceptance was more pronounced in Hospital B, especially given the policy’s low profile. Indeed, Hospital B’s staff seemed to have a stronger tendency to label patients and justify poor behaviour towards them on the grounds of their “unacceptable” or “abusive” behaviour (interview data) and so we judged the hospital’s ethic of care was more weakly institutionalised.

### Explaining policy implementation experiences: The nature of policies; organisational culture, trust and power

In this final section of our results we draw together our analysis of the factors influencing the policy implementation experiences, explicitly considering organisational culture and trust as influences.

#### UPFS

The implementation experiences appear, first, to reflect the different natures of the policies. The UPFS quite clearly spelt out patient categories, requirements for proving income, and the fee level to be charged (Table 1). However, exemption procedures were potentially complicated and cumbersome, requiring patients to produce supporting information (Table 1). This combination supported the revenue generation focus of policy implementation and, since the UPFS was a comparatively unambiguous policy that did not conflict with implementers’ values (unlike the PRC, it did not generate a discourse of grudging acceptance or resistance), it was generally easier to implement. In addition, as already noted, managers in both hospitals supported the policy, often in ways that reinforced the revenue target.

A fuller picture, however, emerges when cross-case comparison allows consideration of the policy’s nature with the inter-connected factors of organisational culture, organisational trust and power.

The UPFS seems to fit key elements of both hospitals’ organisational cultures, in being clearly outlined and having an explicitly determined revenue goal. The rational cultural type is strongly present in both facilities, pointing to competitiveness, achievement and meeting objectives (Figs. 1 and 2). With such values, the revenue goal would be a natural target to aim at, and, indeed, was accepted by all staff. Underpinned by its performance orientation, Hospital A’s senior management had, moreover, attempted to get the revenue target reduced – an action that reflected a concern to secure performance success. Arguably, the revenue target and revenue generation goal had additional significance because they originated with and were important to higher authorities of significance to the hospitals, reflecting the hierarchical elements of both cultures and their emphasis on reporting relationships and adherence to rules and regulations (Figs. 1 and 2).

Despite these similarities, the hospitals had different trust dynamics. Hospital A’s generally more trusting relationships between managers, staff and colleagues (Fig. 3) related to factors such as the clan cultural type, with its premium on participation and inclusion, cohesion and morale (Figs. 1 and 2), reflected in the actions of managers, who role-modelled approachability and inclusive decision-making; as well as the long time that some staff had worked in the facility. The higher organisational trust levels were, therefore, likely related to the positive exercise of managerial power and authority [23]. These factors combined to produce a good stock of trust that would have been fertile ground for cultivating buy-in and ensuring participation in key organisational goals and encouraging different groups such as clerks and medical staff to work together to support UPFS implementation.

The weaker trust relationships of Hospital B (Fig. 4) appeared to derive partly from its strong rational cultural type (Figs. 1 and 2), which is negatively correlated with trust and leader credibility [33] and partly from managerial actions that were perceived to role-model hierarchy and a lack of inclusion. This can be seen, for example, in nurses’ complaints about needing more collective sessions to discuss UPFS implementation and clerks' complaints of feeling under-valued and not being consulted in decisions that affect them (observations and interview data). The weaker trust levels were, therefore, likely related to less productive assertions of managerial power and authority [23].

These dynamics combined to create a diminished stock of trust that likely did not encourage widespread buy-in to organisational goals (e.g. nurses’ lack of cooperation with the case manager) and that fed into tensions across groups about implementation (e.g. some nurses felt the case manager was interfering in ward affairs and struggled to connect with her because of her professional background, and both senior managers and nurses complained that the clerks were not motivated and cooperative enough).

#### PRC

The PRC as a policy was more difficult to implement than the UPFS. It was less clearly specified, comprised multiple dimensions, entailed diffuse activities, and incorporated patient empowerment goals, which suffered from a disjuncture between PRC implementation activities (e.g. posters, suggestion boxes, nametags) and the outcome of better care through a re-balancing of provider-patient relationships (Table 1).

In addition, the PRC to some extent challenged health workers’ values and status, as is evident from the negative staff discourse about the policy (Table 5). These reactions appeared to stem from the PRC’s challenge to providers’ power over patients and emphasis on the inevitable co-production of care, and health workers’ reaction to the often-stressful nature of their work as street-level bureaucrats, including facing patient blame for factors outside their control (observations & interview data). Furthermore, although Hospital A’s managers provided support, management behaviour in Hospital B and the lack of support from higher authorities such as the region, further undermined the PRC’s implementation.

Despite these challenges, both hospitals’ staff largely acted in accordance with the intention of the PRC by demonstrating care and respect for patients. In both hospitals, these positive actions and relationships were underpinned by personal and professional norms, the desire for personal patient appreciation and concern with the hospital’s reputation (observations and interview data). However, the ethic of care was judged to have been more strongly institutionalised in Hospital A than B, where there seemed to be a more diffused responsibility for good provider-patient relationships. This appeared to be related to factors such as the behaviours role-modelled by management, including managers taking action to address patient concerns or going beyond the call of duty in their own work (provider interviews). However, Hospital A’s higher levels of trust in general and trust in management in particular (Fig. 3) was also an element of this mix as it, in line with theory [23], in itself likely generated positive provider-patient relationships in support of the PRC.

The managerial dislike for the PRC in Hospital B and its lack of explicit implementation also suggest a sub-optimal fit between the policy and organisational culture. The hospital’s culture was characterised by the value of order, control and stability (hierarchical and rational: Fig. 2) and organisations such as this can have some difficulty in getting to grips with a policy such as the PRC. It is not very clearly defined, can be interpreted in different ways, seeks to re-balance patient-provider relations and to a large extent relies on the discretion of frontline implementers – all characteristics that might frustrate the desire for control, order and stability.

## Discussion

This research shows that health systems are human systems, with the patient-frontline provider encounter at its core [1]. It illustrates the importance of co-production between provider and patient [38, 39] in policy implementation and shows how this encounter shapes equitable access. It was, therefore, at the interface between the patient and clerk where the UPFS policy was adhered to or not, where decisions influenced patients’ ease of access, and where financial protection was shaped. At the patient-health worker interface relevant to the PRC, meanwhile, interactions might or might not be courteous, and power was negotiated, with decisions made that affected rights such as privacy, confidentiality, treatment refusal, and complaint.

The policies considered had, moreover, long implementation paths embracing chains of relationships between actors [1], starting with a national policy announcement, its diffusion to provincial and service delivery structures and its eventual embodiment in frontline interactions. The essential people-centeredness of this long implementation chain can be described partly in the exercise of individuals’ power, values, ideas and interests across this chain, but also, in how people and relationships were influenced by broader workplace and societal “software”. We, therefore, judge that workplace and provider-patient trust are central to policy implementation, as well as shaping service delivery [2, 23].

However, this research suggests that such “software” is not by definition positive or negative in terms of policy implementation outcomes, including equity, and that these outcomes depend on the contexts and policies in relation to which it is activated. Hospital A and B, for example, were generally well-performing and treated patients well, suggesting a degree of positive, normative people-centeredness in service delivery. Yet, as illustrated by Hospital B and the PRC, the “software” did not fit all policies equally well and left space for managerial resistance to policy implementation. Even where the factors were more supportive, for example the more trusting environment of Hospital A with its positive exercise of managerial power and explicit PRC implementation, there was only a grudging staff acceptance of the policy and negative discourse about patients and how they dealt with their rights and responsibilities, linked to providers’ own understandings of their status and proper behaviour.

It is well-established in public policy that there are different types of policies, that vary in ambiguity, can elicit different stakeholder reactions and that have different implementation requirements [40, 41, 42]. Yet policy implementation remains commonly understood through a top-down lens, as something easily administered not only through multiple relationships, but also across multiple organisational units that are assumed to be similar to each other. Our work challenges this understanding by illuminating the different organisational culture and relationship dynamics in each hospital and their influence over the implementation of two different policies. As Topp et al. found, the “…distinct combination of structural, organisational, relational and cultural components…” in specific Zambian health centres, influenced service responsiveness and quality [43].

What, then, does this study suggest about how to think about managing policy implementation in South Africa and elsewhere? Our work affirms Sheikh et al.’s conclusion: “When we see systems as social institutions primarily defined by the people who constitute them and their human relationships, the ways of bringing about change in health systems go beyond altering written rules and distributing resources, and extend to managing these chains of relationships effectively” [1: ii3].

First, to support policy implementation and organisational adjustment to changing requirements, managers along the implementation chain need to be more aware of “unseen” but important factors such as organisational culture and organisational trust. Managers should understand how these factors can support or hinder change; must have an understanding of themselves as more than mere administrators of policy directives, but as policy implementers with agency who can intervene strategically in this terrain; and must have at least some autonomy to act in contextually sensitive ways [11, 44].

Second, it is necessary to take policy-specific actions. Examples include managerial involvement in the UPFS implementation and allocating resources to the PRC to support strategies like training and poster provision. But it is also important to be careful about how new policies are framed [45]. For example, in both hospitals the meaning of the UPFS centred on revenue generation, highlighting financial metrics and the collection of money and pushing exemptions to the background. Reflecting a specific moment in South African history, the PRC, meanwhile, was framed using the language of rights and responsibilities, and this then invited adversarial behaviours from providers as they compared the rights of health workers and patients – and sometimes led them to judge that patient rights were privileged over their own, and should be conditional on responsibilities. Alternative policy framings, such as improving access, for the UPFS, or quality improvement for the PRC might, instead, have tapped into provider values that supported implementation towards equity goals.

Third, managers must always recognise, and act on, the broader workplace culture created by their actions, in terms of factors like levels of organisational trust, participative management and consultation with staff members. These factors might impact specific policies through staff buy-in or resistance, but are also always important to, for example, the legitimacy of managerial action to support any policy’s implementation.

## Conclusions

Using the conceptual lenses of organisational trust, organisational culture and power to investigate policy implementation, we have highlighted the essential people-centeredness of health systems and the related importance of “software”. This people-centeredness transformed two apparently very similar hospitals into quite different implementation settings.

Achieving equity in practice in South Africa and elsewhere, therefore, requires managers to take account of how an equity-oriented policy might interact with the rich organisational context of its implementation. Such awareness, and associated implementation tasks such as relationship management and the negotiation of values, might be especially important for policies such as the PRC, which can be interpreted as a direct challenge to health workers’ status and values. Achieving equity and people-centered health systems also requires careful attention to how policies are practically framed and translated into practice, with the UPFS providing an example of how the policy itself, its understandings and organisational context can encourage lop-sided implementation.

## Abbreviations

CVF: Competing values framework
LMICs: Low- and middle-income countries
NHI: National Health Insurance
OTI: Organisational trust inventory
PCHS: People-centered health systems
PRC: Patients’ Rights Charter
SLB: Street-level bureaucrats
UHC: Universal Health Coverage
UPFS: Uniform Patient Fee Schedule
